# Dietary Supplementation of Methyl Cedryl Ether Ameliorates Adiposity in High-Fat Diet-Fed Mice

**DOI:** 10.3390/nu15030788

**Published:** 2023-02-03

**Authors:** Mengjie Li, Seong-Gook Kang, Kunlun Huang, Tao Tong

**Affiliations:** 1 Key Laboratory of Precision Nutrition and Food Quality, Key Laboratory of Functional Dairy, Ministry of Education, College of Food Science and Nutritional Engineering, China Agricultural University, Beijing 100083, China; 2 Key Laboratory of Safety Assessment of Genetically Modified Organism (Food Safety), The Ministry of Agriculture and Rural Affairs of the P.R. China, Beijing 100083, China; 3 Beijing Laboratory for Food Quality and Safety, Beijing 100083, China; 4 Department of Food Engineering, Mokpo National University, Muangun 58554, Republic of Korea

**Keywords:** methyl cedryl ether, 16S rRNA sequencing, transcriptome, high-fat diet, obesity

## Abstract

Methyl cedryl ether (MCE) is a derivative of cedrol and is widely used as a fragrance compound. The aim of this study was to evaluate the preventative effects of MCE on obesity and related metabolic syndromes and to delineate the mechanisms from the perspective of gut microbiota and white adipose tissues (WAT) transcriptomic profiles. Five-week-old male C57BL/6J mice were randomly assigned into 3 groups and fed with chow diet, high-fat diet (HFD), or HFD supplemented with 0.2% (*w*/*w*) MCE for 13 weeks. We found that MCE significantly reduced body weight, inhibited adipocyte hypertrophy, and ameliorated hepatic steatosis under HFD conditions. MCE dietary supplementation downregulated the expression of adipogenesis genes (*FAS* and *C/EBPα*) and upregulated the mRNA levels of thermogenesis genes (*PGC-1α*, *PRDM16*, *UCP1*, *Cidea*, *Cytc*, and *COX4*) in epididymal WAT. 16S rRNA sequencing revealed that MCE improved gut microbiota dysbiosis in HFD-fed mice, as manifested by the alteration of strains associated with obesity. Further transcriptome analysis of WAT indicated that MCE dramatically changed the gene expression profiles. Our results demonstrate the anti-obesity effect of MCE under HFD conditions, highlighting the nutraceutical potential of MCE for preventing obesity.

## 1. Introduction

Obesity, traditionally defined as an abnormal expansion of adipose tissue, is causing damage to health [1]. Increased fat deposition is mainly caused by an imbalance between caloric intake and energy expenditure [2]. At this point in time, more than 2 billion people are overweight or obese worldwide, despite targeted public health efforts and treatment measures to combat obesity [3]. Obesity is related to the increasing risk of cardiovascular diseases, type 2 diabetes mellitus, some types of cancer, and other adverse pathological conditions, all of which have a profound impact influence on the life quality [4]. Lifestyle interventions such as diet changes and exercise are the cornerstones of obesity treatment. However, these therapeutic approaches are generally ineffective [5]. Thus, there is still a desperate demand for effective dietary therapies to get obesity under control.

Emerging evidence supports the proposition that gut microbiota is related to the occurrence and development of obesity [6]. The gut microbiota is the community of the various microorganisms that dwell in the intestinal system, and it is tightly associated with the host metabolism [7]. It is shown that gut dysbiosis occurs in obese individuals, manifesting as the remarkable differences of gut microbiota between obese patients and healthy individuals [8]. Various phytochemicals or natural products are demonstrated to be critical obesity regulators by modulating gut microbiota [9,10,11]. As a result, targeting gut microbiota has become a promising option for obesity treatment.

**Figure 1:**
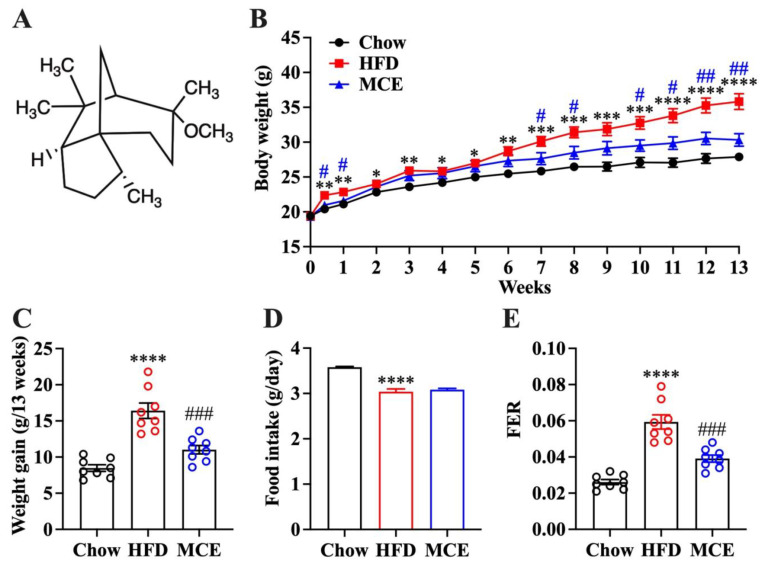
Effects of methyl cedryl ether (MCE) supplementation on high-fat diet (HFD)-induced obesity in mice. (**A**) The chemical structure of MCE. (**B**) Changes in body weight during 13 weeks of feeding. (**C**) Cumulative body weight gain. (**D**) Daily food intake. (**E**) Food efficiency ratio (FER). Data were presented as mean ± SEM (*n* = 8). The data for chow and HFD group mice have been reproduced from ref. [11]. * *p* < 0.05 versus chow group, ** *p* < 0.01 versus chow group, *** *p* < 0.001 versus chow group, **** *p* < 0.0001 versus chow group; # *p* < 0.05 versus HFD group, ## *p* < 0.01 versus HFD group, ### *p* < 0.001 versus HFD group.

Flavor compounds are abundantly present in plants. A large number of evidences show that many flavor compounds possessing good therapeutic potential and safety profile beyond its traditional use as a flavoring agent [12,13]. Methyl cedryl ether (MCE), also recognized as cedramber, is in the structure of terpene (Figure 1A). This derivative is synthesized by methylation of cedrol, which is widely found in the essential oil of conifers and makes up about 19% and 15.8% of cedarwood oil in Texas and Virginia, respectively [14]. MCE has a bright flavor quality between amber and patchouli and is widely used in cosmetic industries (such as soap, detergents, creams, lotions, and perfumes) as a fragrance compound [15,16]. Recently, MCE has been reported to have anticandidal and insecticidal activities [17]. Our team focused on evaluating the beneficial effects of natural flavor compounds in metabolic diseases and previously confirmed that α-cedrene, a structural analog with MCE, exhibited excellent protective effects on obesity and hepatic steatosis in HFD-fed mice [18,19]. Nevertheless, the protective effects of MCE on obesity and related metabolic syndromes have not yet been investigated. It is still unclear whether it is related to the changes in gut microbiota.

The aim of the present study was to evaluate the potential of dietary MCE supplementation against obesity in high-fat diet (HFD)-fed mice and to clarify the involved mechanisms with attention to gut microbiota composition and white adipose tissues (WAT) transcriptome profiling.

## 2. Materials and Methods

### 2.1. Materials

MCE (96% pure) was obtained from Aladdin Biochemical Technology Co., Ltd. (Shanghai, China). Low-density lipoprotein cholesterol (LDL-C), high-density lipoprotein cholesterol (HDL-C), and total cholesterol (TC) biochemical parameters kits were purchased from Biosino Bio-Technology and Science (Beijing, China).

### 2.2. Animals and Designs

Male C57BL/6J mice (4-week-old) were purchased from Vital River Laboratories (Beijing, China) and housed in specific pathogen-free conditions (12 h light-dark cycle, humidity ranging from 40–70%, and temperature 22 ± 2 °C).

After acclimatization for 1 week, mice were randomly divided into three groups: chow, HFD, and MCE groups (*n* = 8 per group). A standard chow diet (Huafukang, Beijing, China) was administered to the mice in the chow group. As previously reported, HFD was prepared with 40% of calories derived from fat (Table 1) [20]. The HFD supplemented with 0.2% (*w*/*w*) MCE (equivalent to 200 mg/kg) was used to fed mice in the MCE group. During the 13 week long experiment, animal body weight was recorded weekly and food intake was measured daily. Food efficiency ratio (FER) was calculated as body weight gain (g)/total food consumption (g). After 13 weeks, fresh feces were collected and the mice were sacrificed following a 6 h morning fast. The blood was sampled and centrifuged to obtain serum. The WAT and liver were carefully dissected, weighed, and stored at −80 °C for subsequent investigation.

The China Agricultural University Laboratory Animal Welfare and Animal Experimental Ethical Committee authorized all procedures used in this investigation (AW40201202-4-8, Beijing, China).

### 2.3. Fasting Blood Glucose (FBG) and Biochemical Parameters Analysis

The FBG was performed on 6 h morning fast mice 1 week before the end of the experiment. Biochemical parameters kits (Biosino Bio-Technology and Science, Beijing, China) and an Inkiko automatic biochemical analyzer (Thermo Fisher, Waltham, MA, USA) were used to analysis the serum LDL-C, HDL-C, and TC concentrations.

### 2.4. Histological Examination

As previously described, histological examinations of epididymal WAT and liver were conducted by hematoxylin-eosin (H & E) staining, and the image was analyzed using Image J software (version 1.53) [11].

### 2.5. Analysis of Hepatic Triglyceride (TG) and TC Content

For measurement of hepatic TG and TC content, ethanol absolute was added to the accurately weighed liver tissue at a ratio of 1:9 (g/mL), followed by homogenization and centrifuge at 2500 rpm for 10 min. The TG and TC contents of the supernatant were determined using a triglyceride assay kit and a total cholesterol assay kit (Nanjing Jiancheng Bioengineering Institute, Nanjing, China).

### 2.6. Real-Time Quantitative PCR (RT-qPCR)

Total RNA from epididymal WAT was extracted using TRIzol^®^ Reagent (Invitrogen) as directed by the manufacturer, and the cDNA was synthesized using a reverse transcription kit (TIANGEN, Beijing, China). RT-qPCR was performed on a CFX96 Touch Real-Time PCR Detection System (Bio-Rad, Hercules, CA, USA) by using SYBER Green Supermix (TIANGEN, Beijing, China). The mRNA expression was normalized using *GAPDH* expression. The primer sequences were presented in Table S1.

### 2.7. Gut Microbiota Analysis

Total fecal DNA was extracted according to previously reported methods [21]. The quality of all DNA samples was tested by using NanoDrop (Thermo Fisher Scientific, Waltham, MA, USA) and agarose gel electrophoresis. The V3-V4 region of 16S rRNA was amplified from DNA, and the paired-end sequencing of amplicons was performed on the Illumina MiSeq sequencing platform. QIIME (version 2020.2) was used to evaluate alpha and beta diversities. The Majorbio Cloud (Shanghai, China) was utilized to build visualizations of principal coordinates analysis (PCoA), to conduct the linear discriminant analysis effect size (LEfSe) analysis with filters (*p* < 0.05 and LDA score > 4), and to perform Pearson correlation analysis. The predicted functional profiles of microbial communities were conducted using PICRUSt, and the statistically significant differences were evaluated in STAMP version 2.1.3 (http://kiwi.cs.dal.ca/Software/STAMP (accessed on 25 July 2022)).

### 2.8. Epididymal WAT Transcriptome Analysis

Total RNA from epididymal WAT was extracted as described above. RNA concentration and purity were determined using an ND-2000 (NanoDrop Technologies, Wilmington, DE, USA) and a 2100 Bioanalyser (Agilent Technologies, Santa Clara, CA, USA), respectively. The TruSeqTM RNA sample preparation kit (San Diego, CA, USA) from Illumina was used to build the library of RNA-seq transcriptome. Paired-end RNA-seq sequencing was undertaken using an Illumina HiSeq X ten/NovaSeq 6000 sequencer, yielding 150 bp reads. Quality clipping and adapter trimming of the raw reads were performed with SeqPrep and Sickle. Read mapping was performed with StringTie. DESeq2 was utilized to identify the differential expression genes (DEGs), and the significance was set as |log2FC| > 1 and *p*-adjust < 0.05. The Majorbio Cloud (Shanghai, China) was utilized to build visualizations of principal coordinates analysis (PCoA). Gene-set enrichment analysis was performed using KOBAS by interrogating the pathways in the Kyoto Encyclopedia of Genes and Genomes (KEGG) databases.

### 2.9. Association Analysis of Gut Microbiota and DEGs

Pearson correlation was performed to analysis the association between the DEGs belonging to the PPAR signaling pathway and the genus-level gut microbiota (Top 30). Cytoscape version 3.9.1 (https://cytoscape.org/ (accessed on 12 October 2022)) was used to visualize the significant gene-microbe correlations (*p* < 0.05) as a network.

### 2.10. Statistical Analysis

All data were shown as means ± SEM. Data analysis was conducted using unpaired two-tailed Student’s *t*-test in GraphPad Prism 9.0 (GraphPad Software, Inc., San Diego, CA, USA), and statistically significance was considered as *p* < 0.05.

## 3. Results

### 3.1. MCE Has a Strong Preventive Potential against HFD-Induced Body Weight Gain in Mice

In the HFD-fed mice, MCE dietary supplementation significantly reduced the body weight after feeding for one week, and the final body weight was comparable to the chow group (Figure 1B). MCE supplementation for 13 weeks notably decreased HFD-induced weight gain in mice (Figure 1C). No remarkable difference in food intake between the mice of MCE and HFD group was observed, suggesting that MCE supplementation protects mice from HFD-induced obesity without a decrease in food consumption (Figure 1D). The FER of the MCE group mice was significantly decreased compared to that of HFD group mice (Figure 1E).

### 3.2. MCE Lowers FBG and Improves Serum Lipid Profile in HFD-Fed Mice

The FBG level in MCE-supplemented mice was remarkably lower than that of HFD group mice and was comparable to that of chow diet-fed mice (Table 2). In mice fed with HFD, the effect of MCE on glucose tolerance was minimal (data not shown). Meanwhile, we observed that the serum lipid profile was improved during MCE treatment in HFD-fed mice (Table 2).

### 3.3. MCE Attenuates the Adipocyte Hypertrophy and Hepatic Steatosis in HFD-Fed Mice

**Figure 2:**
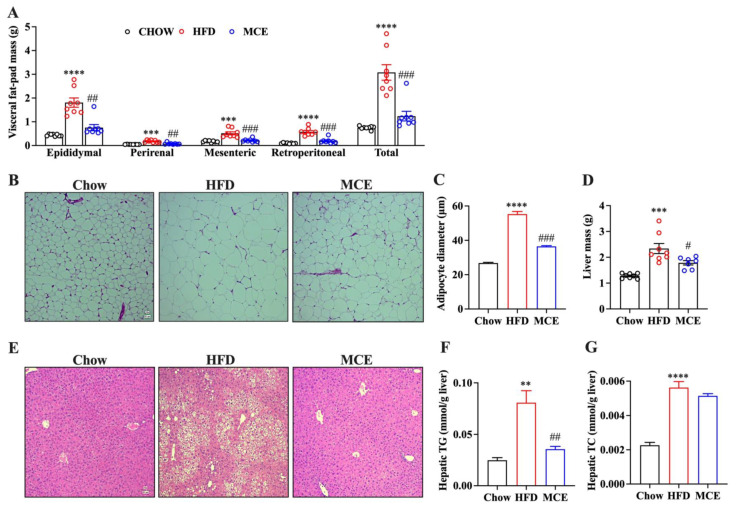
Effects of MCE on the hypertrophy of white adipose tissues (WAT) and hepatic steatosis in HFD-fed mice. (**A**) Visceral fat pad weights. (**B**) Representative images of H&E-stained sections of epididymal adipose tissue (100×). (**C**) Quantification of adipocyte diameter. (**D**) Liver mass. (**E**) Representative images of H&E-stained sections of liver tissue (100×). (**F**) Hepatic TG content. (**G**) Hepatic TC content. ** *p* < 0.01 versus chow group, *** *p* < 0.001 versus chow group, **** *p* < 0.0001 versus chow group; # *p* < 0.05 versus HFD group, ## *p* < 0.01 versus HFD group, ### *p* < 0.001 versus HFD group.

Excess visceral adipose tissue accumulation is an important pathological phenotype related to obesity [22]. Here, we observed a significant decrease in the visceral fat-pad weight in MCE-supplemented mice in comparison to that of HFD group mice (Figure 2A). Next, we conducted H&E staining and analyzed the histology and pathology changes of epididymal adipose tissue, and the results revealed that the HFD-induced hypertrophy of adipocytes was significantly improved with MCE supplementation (Figure 2B). Image J measurements showed that the average adipocyte diameter of MCE group mice was 36.46 μm, which was significantly lower than that of HFD mice (55.29 μm) (Figure 2C).

Long term HFD is one of the main causes of hepatic steatosis and can further develop into nonalcoholic fatty liver disease. As shown in Figure 2D, MCE supplementation for 13 weeks remarkably decreased liver mass in HFD-fed mice. The histological examination by H & E staining indicated that 13 weeks HFD feeding induced obvious lipid deposition in the liver, which was markedly attenuated by MCE supplementation (Figure 2E). In addition, excessive HFD consumption increased liver TG and TC contents in mice, and MCE supplementation significantly decreased the liver TG content in HFD-fed mice whilst having no remarkable effect on liver TC content (Figure 2F,G).

### 3.4. MCE Alters the Expression of Genes Involved in Adipogenesis and Thermogenesis in Epididymal WAT

**Figure 3:**
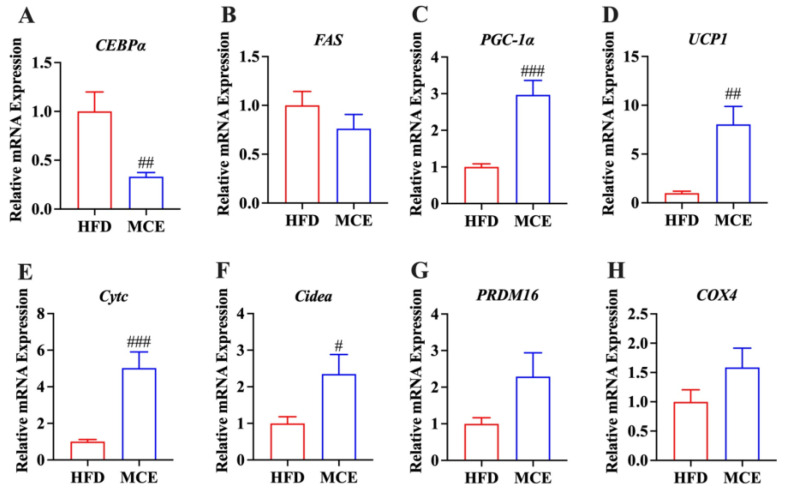
MCE alters the expression of genes related to adipogenesis and thermogenesis in epididymal WAT. The expression of (**A**–**C**) adipogenesis and (**D**–**H**) thermogenic genes in the epididymal adipose tissues of mice. CCAAT/enhancer binding-protein α (*C/EBPα*). Fatty acid synthase (*FAS*). Peroxisome proliferator-activated receptor gamma coactivator 1-alpha (*PGC-1α*). Uncoupling protein 1 (*UCP1*). Cytochrome c (*Cytc*). Cell death activator CIDE-A (*Cidea*). PR domain containing 16 (*PRDM16*). Cytochrome c oxidase subunit 4 (*COX4*). # *p* < 0.05 versus HFD group, ## *p* < 0.01 versus HFD group, ### *p* < 0.001 versus HFD group.

We detected whether MCE supplementation modified the mRNA levels of molecules related to adipogenesis and thermogenesis in the epididymal WAT of mice. The results revealed that MCE downregulated the expression of *FAS* and *C/EBPα* relative to that of HFD group (Figure 3A,B). In parallel, the expression of mitochondrial and thermogenic genes, including *PGC-1α*, *PRDM16*, *UCP1*, *Cidea*, *Cytc*, and *COX4*, were upregulated by MCE supplementation in comparation with those of HFD group mice (Figure 3C–H).

### 3.5. MCE-Supplemented Mice Show Apparent Changes in Gut Microbial Structure

**Figure 4:**
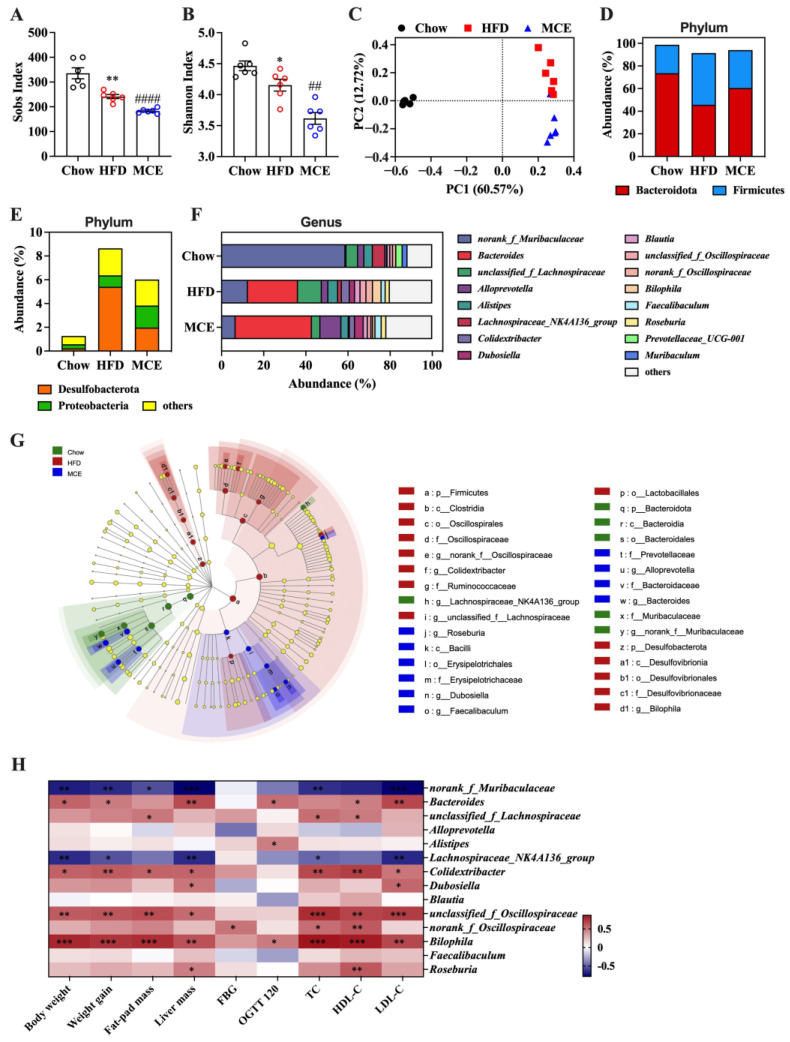
MCE supplementation modulates the gut microbiota in HFD-fed mice. The α-diversity is represented by the (**A**) Sobs index and (**B**) Shannon index. * *p* < 0.05 versus chow group, ** *p* < 0.01 versus chow group; ## *p* < 0.01 versus HFD group, #### *p* < 0.0001 versus HFD group. (**C**) The principal coordinate analysis (PCoA) based on Bray-Curtis distance. The relative abundance of gut microbiota at (**D**), (**E**) phylum level and (**F**) genus level. (**G**) The linear discriminant analysis effect size (LEfSe) analysis. (**H**) Pearson correlation analysis between obesity-related indexes and relative abundance of gut microbiota at genus level. The blue color represents a negative correlation, while the red color represents a positive correlation. The color depth is proportional to the degree of the correlation. * *p* < 0.05, ** *p* < 0.01, *** *p* < 0.001.

After this, we further explored the effect of MCE supplementation on the structure and composition of gut microbiota in HFD-fed mice using 16S rRNA sequencing. Alpha diversity (Sobs and Shannon index) was impacted by MCE treatment in mice fed with HFD (Figure 4A,B). PCoA showed an apparent separation between the HFD and MCE groups, indicating that MCE supplementation had a significant effect on the gut microbiota structure in HFD-fed mice (Figure 4C).

At the phylum level, Bacteroidota, Firmicutes, Desulfobacterota, and Proteobacteria were the predominant phylum in the gut microbiota (Figure 4D,E). HFD feeding decreased the abundance of Bacteroidota, and increased the abundance of Firmicutes, Desulfobacterota, and Proteobacteria with a higher ratio of Firmicutes to Bacteroidota (F/B) (Figure S1). The abundance of Desulfobacterota was particularly decreased in response to MCE administration (Figure S1). In addition, the levels of Bacteroidota, Firmicutes, Proteobacteria, and F/B were also affected by MCE, even though no remarkable difference was observed (Figure S1). At the genus level, the gut microbiota was dominated by *norank_f_Muribaculaceae* and *Bacteroides* (Figure 4F). We found that *Alloprevotella* was dramatically enriched by MCE supplementation in HFD-fed mice. Moreover, the significantly decreased abundance of *norank_f_Muribaculaceae*, *Bilophila, unclassified_f_Lachnospiraceae*, *Lachnospiraceae_NK4A136_group*, *unclassified_f_Oscillospiraceae*, and *norank_f_Oscillospiraceae* was observed in MCE group mice in comparation with those of HFD group mice (Figure S1). Differentially enriched fecal bacterial taxa from phylum to genus in chow, HFD, and MCE groups were identified by LEfSe analysis (Figure 4G).

Pearson correlation analysis was further conducted to understand the correlation between the abundance of microbes at genus level and obesity-related index (Figure 4H). The results revealed that the relative abundance of *unclassified_f_Lachnospiraceae*, *Colidextribacter*, *unclassified_f_Oscillospiraceae*, *norank_f_Oscillospiraceae*, and *Bilophila* were significantly and positively associated with the obesity-related index. Moreover, the relative abundance of the bacteria *Lachnospiraceae_NK4A136_group* and *norank_f_Muribaculaceae* were decreased in the MCE group and were significantly and negatively correlated with the obesity-related index.

Overall, alteration of the gut microbiota in HFD-fed mice occurred in response to MCE administration, suggesting that the gut microbiota might play a critical role in alleviating HFD-induced obesity using MCE.

### 3.6. Predicted Metabolic Profiles of the Gut Microbiota after MCE Supplementation

**Figure 5:**
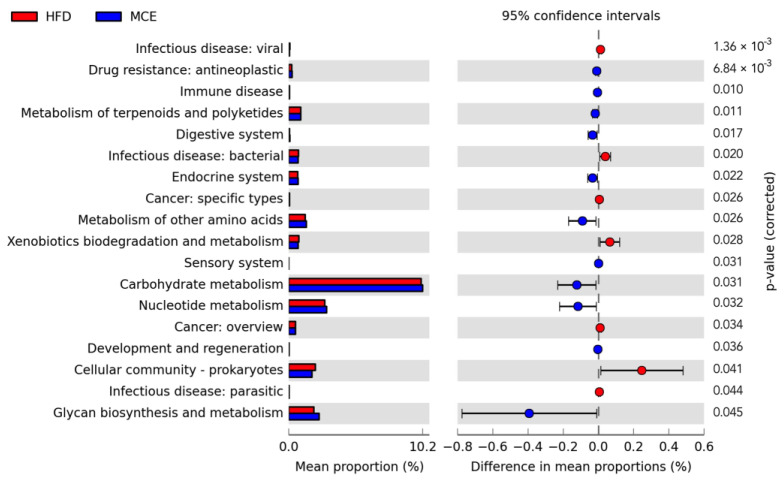
Predicted metabolic profiles of the gut microbiota after MCE supplementation in HFD-fed mice. Statistical significance difference was assessed using the unpaired two-tailed Student’s *t*-test (*p* < 0.05) in STAMP.

The predicted metabolic profiles of the gut microbiota were performed by PICRUSt based on the 16S rRNA sequencing, and the results indicated that multiple pathways were dramatically altered after MCE supplementation in HFD-fed mice. As shown in Figure 5, MCE supplementation greatly boosted the metabolism of terpenoids and polyketides, nucleotide, and other amino acids in comparison with that of HFD group mice. Ccancer, infectious disease, and xenobiotics biodegradation and metabolism were more likely to be inhibited by MCE supplementation. Moreover, the gut microbiota altered by MCE supplementation was predicted to improve drug resistance, digestive system, endocrine system, sensory system, carbohydrate metabolism, and glycan biosynthesis and metabolism in HFD-fed mice.

### 3.7. MCE Supplementation Modifies the Epididymal WAT Transcriptome in HFD-Fed Mice

**Figure 6:**
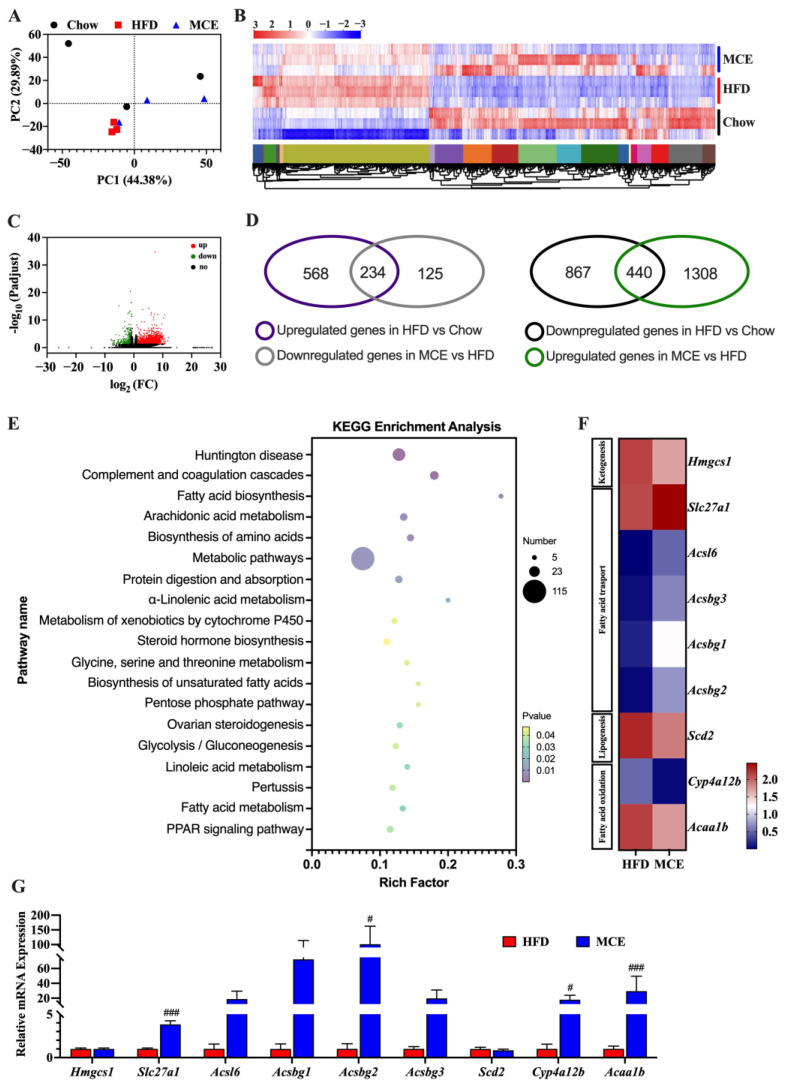
MCE modifies the transcriptome profile of epididymal WAT in HFD-fed mice. (**A**) Principal component analysis of the epididymal WAT Transcriptome. (**B**) Hierarchical clustering of RNA-seq data from epididymal WAT samples is shown using a heatmap. (**C**) Volcano plots indicate the differential expression genes (DEGs) in the epididymal WAT between the HFD and MCE group. (**D**) Venn diagrams indicate the DEGs among chow, HFD, and MCE groups. (**E**) Enriched KEGG analysis of DEGs between HFD and MCE groups (*p* < 0.05). (**F**) Heatmap of the DEGs between HFD and MCE groups involved in the PPAR signaling pathway. *n* = 3. (**G**) mRNA levels of *Hmgcs1*, *Scd2*, *Cyp4a12b*, *Acaa1b*, *Acsbg1*, *Acsbg2*, *Acsbg3*, *Slc27a1*, and *Acsl6* in epididymal WAT tested by RT-qPCR. *n* = 8. # *p* < 0.05 versus HFD group, ### *p* < 0.001 versus HFD group.

Next, we performed RNA sequencing in the epididymal WAT to uncover the underlying mechanisms of the protective potential of MCE supplementation in HFD-fed mice. The PCA showed obviously separate clustering of the three groups (Figure 6A). Consistent with the PCA results, the hierarchical clustering of the WAT transcriptome revealed distinct transcriptional profiles for the HFD and MCE groups (Figure 6B). A certain similarity in transcriptional profiles was observed between the chow and MCE groups (Figure 6B). In addition, we found that 1748 genes were up-regulated and 359 genes were down-regulated in the MCE group in comparation with that of HFD group.

Based on all 2107 DEGs between HFD and MCE groups, we further carried out KEGG enrichment analysis to identify the major molecular pathways and gene functions. As shown in Figure 6E, several DEGs were considerably enriched in metabolic pathways known to be associated with obesity, including carbohydrate metabolism (pentose phosphate pathway and glycolysis/gluconeogenesis) and lipid metabolism (linoleic acid metabolism, biosynthesis of unsaturated fatty acids, steroid hormone biosynthesis, alpha-linolenic acid metabolism, arachidonic acid metabolism, and fatty acid biosynthesis). Moreover, compared to the HFD group, KEGG enrichment analysis revealed that the genes involved with the PPAR signaling pathway were remarkably regulated in the MCE group. Moreover, compared to the HFD group, KEGG enrichment analysis revealed that the genes involved with the PPAR signaling pathway were remarkably regulated in the MCE group. Congruent with the data of RNA-seq, the RT-qPCR analysis verified that the expression of *Slc27a1* and *Acsbg2* in the MCE group were significantly higher than those of HFD group (Figure 6G). Nonetheless, MCE significantly upregulated the expression of Cyp4a12b and Acaa1b in HFD-fed mice, which contradicted the results of transcriptome analysis (Figure 6F,G).

### 3.8. Interactions between Gene Expression Profile in WAT and Gut Microbiota

**Figure 7:**
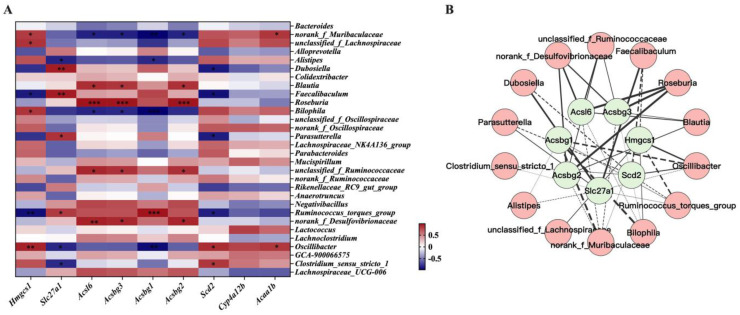
Correlation analysis of DEGs change and gut microbiota alterations. (**A**) Correlation heatmap between enriched DEGs determined by RNA-seq and gut microbiota at the genus level. The red color represents a positive correlation, while the blue color represents a negative correlation. The color depth is proportional to the degree of the correlation. * *p* < 0.05, ** *p* < 0.01, *** *p* < 0.001. (**B**) Network visualizing significant gene-microbe correlations. Green represents enriched DEGs and pink represents microorganisms. The solid lines represent a positive relationship, while the dashed lines represent a negative relationship. Edge thickness represents the strength of the correlation.

To investigate host-microbe relationships further, we considered correlations between 9 DEGs enriched to the PPAR signaling pathway, on the one hand, and the top 30 gut microbial taxa at genus level, on the other hand. As shown in Figure 7A, Pearson correlation analysis revealed 42 significant unique gene-microbe correlations. We visualized these significant correlations between host gene expression and taxa abundance using Cytoscape (Figure 7B). In particular, our findings demonstrated that the abundance of *norank_f_Muribaculaceae*, *Bilophila*, and *Oscillibacter* exerted a negative relationship with the expression level of *Acsbg1*, and the relative abundance of *Dubosiella* and *Faecaliabaculum* was positively associated with the expression level of *Slc27a1*. In addition, the relative abundance of *norank_f_Desulfovibrionaceae* and *Roseburia* was positively related to the expression of *Acsbg2*, *Acsbg3*, and *Acsl6*.

## 4. Discussion

Recently, the role of many natural flavor compounds has been repositioned, which means that they have been found to have a variety of nutraceutical and therapeutic potential effects beyond their traditional use as flavoring agents [13]. For example, vanillic acid, eugenol, and many other famous fragrant materials have been demonstrated to have pharmacological activities. In addition to the anticandidal and insecticidal activities in vitro [17], the protective effects of MCE on metabolic diseases are still unclear. In order to extend the application of MCE in the biomedicine field, in this study we conducted an animal experiment and found that MCE exhibited a favorable effect on adiposity at a dose of 200 mg/kg without evident toxicity. The dosage used in the present study is equivalent to 0.97 g/day for a 60 kg adult human, which provides the ability to translate our findings into the human treatment [23]. The acute toxicity test of MCE indicated that both the acute dermal LD_50_ in rats and the acute oral LD_50_ in rabbits were greater than 5 g/kg [24]. However, to our knowledge, there are no studies on the chronic toxicity of MCE so far. Despite the fact that our tests did not extensively examine the safety of MCE, no mortality or treatment-related adverse clinical indications were observed during the 13 week experiment. Before the medicinal use of MCE is examined in humans, extensive safety investigations will be required.

Targeting gut microbiota is a viable preventative or therapeutic approach for obesity due to the link between gut bacteria and obesity [7,25]. Curcumin, betaine, garcinol, eugenol, and other dietary components appear to alleviate obesity by modifying the gut flora [11,26,27,28]. In the current study, 13 weeks of MCE treatment prevents HFD-induced obesity in mice while also modulating the structure, composition, and metabolic profiles of gut microbiota (Figure 4 and Figure 5). It has been reported that Firmicutes and Bacteroidota in the gut are highly linked to the regulation of the host energy homeostasis. Obesity and related metabolic syndromes may be exacerbated by a higher ratio of Firmicutes but a lower ratio of Bacteroidota in the gut [29,30]. Similarly, we found that HFD-fed mice had a larger abundance of Firmicutes and a lower abundance of Bacteroidetes than chow-fed mice. In addition, a reduced F/B was observed in the MCE group versus the HFD group, though the difference was not significant (Figure S1). Desulfobacterota is well-known for its capacity to reduce sulfate, and it also exhibits aliphatic and aromatic hydrocarbon degradation and nitrogen fixation abilities [31]. Here, we found that MCE treatment significantly tracked to an decreased abundance of Desulfobacterota in HFD-fed mice.

To date, the relationship between obesity and specific taxa have been widely investigated. *Alloprevotella* is well known as a beneficial bacteria, and Cai et al. [32]. pointed out that lower abundance levels of *Alloprevotella* are the main characteristic of gut dysbiosis in *db*/*db* mice, which could be reversed by resveratrol treatment. *Bilophila*, a kind of bile-tolerant microorganism, belongs to the phylum Desulfobacterota and is positively associated with the progression of obesity [33]. Furthermore, obesity-related indicators have been reported to be positively connected with the abundances of *unclassified_f_Lachnospiraceae*, *unclassified_f_Oscillospiraceae*, and *norank_f_Oscillospiraceae* [11,34]. Our results consistently revealed that *Alloprevotella* was enriched by MCE supplementation, and a significant decrease in abundance of *Bilophila, unclassified_f_Lachnospiraceae*, *unclassified_f_Oscillospiraceae*, and *norank_f_Oscillospiraceae* was observed in the MCE group compared to the HFD group (Figure S1). Moreover, the abundance of *norank_f_Muribaculaceae* and *Lachnospiraceae_NK4A136_group* has been believed to be negatively associated with the development of obesity [35,36]. However, in the present study, we observed that the supplementation of MCE induced a decreased abundance of *norank_f_Muribaculaceae* and *Lachnospiraceae_NK4A136_group*. Thus, more investigation into the significance of these two microorganisms in the preventative impact of MCE on adiposity is required. Taken together, the anti-obesity effect of MCE appears to be ascribed in part to the improvement of gut microbiota; however, further research in germ-free mice or antibiotic-treated mice is necessary to address this issue.

WAT comprises the bulk of body fat and is an important location for energy balance, insulin signaling, and endocrine function [37]. To enhance preadipocyte development into mature adipocytes, which are the major cells in adipose tissue, white adipocytes require crucial transcription factors [38]. *C/EBPα*, the genes encoding a transcription factor, is considered a critical early regulator of adipogenesis and lipid storage in adipocytes. At the same time, its target, *FAS*, is responsible for forming mature adipocytes [39]. Mounting evidence indicates that the administration of natural agents alleviates obesity by downregulating *C/EBPα* and *FAS* gene expression in HFD-fed mice [40,41]. *PGC-1α* is a critical transcriptional coactivator and induces the transcription of its downstream thermogenic (*PRDM16*, *Cidea*, and *UCP1*) and mitochondrial (*Cytc* and *COX4*) genes in white adipocytes [42,43]. It has been confirmed that the increased expression of *PGC-1α* in WAT is critical in combatting obesity [44]. In this study, MCE treatment downregulated the mRNA expression of *C/EBPα* and FAS and upregulated the mRNA expression of *PGC-1α*, *PRDM16*, *Cidea*, *UCP1*, *Cytc,* and *COX4* in HFD-fed mice (Figure 3), indicating that the MCE-induced decrease in body weight was associated with the regulation of molecules related to adipogenesis and thermogenesis in epididymal WAT.

Obesity and insulin resistance are linked to an increase in epididymal adipocytes [42]. Herein, we further analyzed the epididymal WAT transcriptome to explore the underlying mechanism of the observed phenotype. We found that the PPAR signaling pathway was strongly affected by MCE supplementation as compared to the HFD group (Figure 6E). The expression of genes related to fatty acid oxidation (*Cyp4a12b* and *Acaa1b*) and fatty acid transport (*Slc27a1* and *Acsbg2*) were extremely upregulated by MCE (Figure 6F,G). Recently, Guo et al. [45] demonstrated that the preventative effect of Withaferin A on HFD-induced obesity was related to the upregulated expression levels of *Slc27a1* in WAT. *Cyp4a12b*, a member of the cytochrome P450 family, is an essential factor related to lipid oxidation levels and was decreased by HFD and increased by kaempferol treatment in mice [46]. In addition, *Acaa1b*, belonging to the thiolase family of enzymes, catalyzes the final step of β-oxidation [47]. In sum, the preventative effect of MCE against HFD-induced epididymal adipocyte accumulation was linked to the regulation of the PPAR signaling pathway.

Typical features of hepatic steatosis, including increases in the lipid content and degree of liver impairment, can also be observed during the progression of obesity [48]. The expansion of adipose tissue in obesity diminishes its ability to store excess energy. Consequently, adipocyte dysfunction is increased, leading to fatty deposits in the liver [49]. The features of hepatic steatosis were alleviated by MCE treatment, as demonstrated by the improved mass and fat deposition in liver of HFD-fed mice (Figure 4D,E). Thus, the alleviation of hepatic steatosis in HFD-fed mice could also be a secondary event followed by the improvement of obesity.

## 5. Conclusions

In summary, the present study demonstrates that dietary supplementation of MCE is very effective in controlling obesity and related metabolic disorders in HFD-fed mice. In epididymal WAT, the expression of molecules related to adipogenesis and thermogenesis was remarkably regulated by MCE. The protective effect of MCE is linked to the alteration of gut microbiota composition and WAT transcriptomic profiles. These results provide the basis for utilising MCE to prevent HFD-induced obesity and gut dysbiosis.

## Figures and Tables

**Table 1:** Composition of the experimental diets (g/kg) fed to mice.

Ingredients	HFD Group	MCE Group
Casein	200	200
DL-Methionine	3	3
Corn starch	111	109
Sucrose	370	370
Cellulose	50	50
Corn oil	30	30
Lard	170	170
Mineral mixture ^a^	42	42
Vitamin mixture ^b^	12	12
Choline bitartrate	2	2
Cholesterol	10	10
Methyl cedryl ether	-	2
tert-Butylhydroquinone c	0.04	0.04
Fat, % kJ	40	40

^a^ Mineral mixture for AIN-76A rodent diet. ^b^ Vitamin mixture for AIN-76A rodent diet. HFD, high-fat diet; MCE, methyl cedryl ether.

**Table 2:** Effects of MCE supplementation on FBG and serum lipid profiles in HFD-fed mice.

	Chow	HFD	MCE
FBG (mmol/L)	8.41 ± 0.22	10.01 ± 0.46 **	7.68 ± 0.51 ^##^
TC (mmol/L)	2.83 ± 0.08	7.30 ± 0.31 ****	4.29 ± 0.16 ^####^
HDL-C (mmol/L)	2.05 ± 0.07	3.57 ± 0.11 ****	2.68 ± 0.14 ^###^
LDL-C (mmol/L)	0.34 ± 0.01	1.65 ± 0.16 ***	1.23 ± 0.12 ^#^
HDL-C/LDL-C	6.03 ± 0.24	2.33 ± 0.24 ***	2.35 ± 0.28

Data were presented as mean ± SEM (*n* = 8). FBG, fasting blood glucose; TC, total cholesterol; HDL-C, high-density lipoprotein cholesterol; LDL-C, low-density lipoprotein cholesterol. ** *p* < 0.01 versus chow group, *** *p* < 0.001 versus chow group, **** *p* < 0.0001 versus chow group; ^#^ *p* < 0.05 versus HFD group, ^##^ *p* < 0.01 versus HFD group, ^###^ *p* < 0.001 versus HFD group, ^####^ *p* < 0.0001 versus HFD group.

## Data Availability

The data that support the findings of this study are available from the corresponding author upon reasonable request.

## Supplementary Materials

The following supporting information can be downloaded at: https://www.mdpi.com/article/10.3390/nu15030788/s1, Table S1: Primer sequences used for RT-qPCR; Figure S1: Relative abundance of gut microbiota at phylum and genus levels.
