# Supplementary material for: Dietary Supplementation of Methyl Cedryl Ether Ameliorates Adiposity in High-Fat Diet-Fed Mice
Source: Nutrients. 2023 Feb 3;15(3):788. doi: 10.3390/nu15030788 (PMC9919899; doi:10.3390/nu15030788)
Supplement: Supplementary file 1 [file nutrients-15-00788-s001.zip › nutrients-2082420-supplementary.pdf]

Supporting Information

Dietary supplementation of methyl cedryl ether ameliorates adiposity in high-fat diet-fed mice

Mengjie Li [1,2,3], Seong-Gook Kang [4], Kunlun Huang [1,2,3] and Tao Tong [1,2,3,*]

[1] Key Laboratory of Precision Nutrition and Food Quality, Key Laboratory of Functional Dairy, Ministry of Education, College of Food Science and Nutritional Engineering, China Agricultural University, Beijing 100083, China
[2] Key Laboratory of Safety Assessment of Genetically Modified Organism (Food Safety), The Ministry of Agriculture and Rural Affairs of the P.R. China, Beijing 100083, China
[3] Beijing Laboratory for Food Quality and Safety, Beijing 100083, China
[4] Department of Food Engineering, Mokpo National University, Muangun 58554, Republic of Korea
* Correspondence: tongtao1028@cau.edu.cn; Tel.: +86-10-6273-7244

Table S1. Primer sequences used for RT-qPCR.

Type	Gene Description	Sequences (5'→3')
Mouse	Peroxisome proliferator-activated receptor γ2 (*PPARγ2*)	F: TTCGGAATCAGCTCTGTGGA<br>R: CCATTGGGTCAGCTCTTGTG
	CCAAT/enhancer binding-protein α (*C/EBPα*)	F: TCAGCTTACAACAGGCCAGG<br>R: ACACAAGGCTAATGGTCCCC
	Fatty acid synthase (*FAS*)	F: TTGCTGGCACTACAGAATGC<br>R: AACAGCCTCAGAGCGACAAT
	Peroxisome proliferator-activated receptor gamma coactivator 1-alpha (*PGC-1α*)	F: TAAATCTGCGGGATGATGGA<br>R: GTTTCGTTCGACCTGCGTAA
	Uncoupling protein 1 (*UCP1*)	F: GGTTTGCACCACACTCCTG<br>R: ACATGGACATCGCACAGCTT
	PR domain containing 16 (*PRDM16*)	F: CCACCAGCGAGGACTTCAC<br>R: GGAGGACTCTCGTAGCTCGAA
	Cytochrome c (*Cytc*)	F: ACACTGTGGAAAAGGGAGGC<br>R: GCACTGGTTAACCCAAGCAA
	Cytochrome c oxidase subunit 4 (*COX4*)	F: GTACCGCATCCAGTTTAACGA<br>R: CCATACACATAGCTCTTCTCCCA
	Cell death activator CIDE-A (*Cidea*)	F: GGAATCTGCTGAGGTTTATG<br>R: ATCCCACAGCCTATAACAGA
	3-hydroxy-3-methylglutaryl-Coenzyme A synthase 1 (*Hmgcs1*)	F: GCCGTGAACTGGGTCGAA<br>R: GCATATATAGCAATGTCTCCTGCAA
	stearoyl-Coenzyme A desaturase 2 (*Scd2*)	F: AGTATTGCCACCCAGATGCT<br>R: GTGGGGCACGAACCTTTACT

cytochrome P450 family 4 subfamily A polypeptide 12B (*Cyp4a12b*)	F: CCACTCATTCCTGCCCTTC<br>R: TCAGCTCATTCATCGCAAAC
acetyl-Coenzyme A acyltransferase 1B (*Acaa1b*)	F: ATGGGAATATTTCTTCCCGC<br>R: ATTAAAGTGCTGTGACACAC
solute carrier family 27 member 1 (*Slc27a1*)	F: AGCCTGGTCAAGTTCTGTTCTGGA<br>R: AGAAGAGTCGATCATCCATGCCCT
acyl-CoA synthetase long-chain family member 6 (*Acsl6*)	F: CAGAGGAACTCAACTACTGGACC<br>R: CCAATGTCTCCAGTGTGAAGCC
acyl-CoA synthetase bubblegum family member 1 (*Acsbg1*)	F: ACTCGCAAACCAGCTCCTT<br>R: CCGGGTTGTCCATAGTGCTT
acyl-CoA synthetase bubblegum family member 2 (*Acsbg2*)	F: ATGCCTCCAACAGATCAGCC<br>R: TGTTTTGGGCTCTGGGTGTT
acyl-CoA synthetase bubblegum family member 3 (*Acsbg3*)	F: AACCCGTATCCCATCGAGGA<br>R: CTTCGAAGCTTCCGACAGCA
Glyceraldehyde-3-phosphate dehydrogenase (*GAPDH*)	F: GTGATGGCATGGACTGTGGT<br>R: GGAGCCAAAAGGGTCATCAT

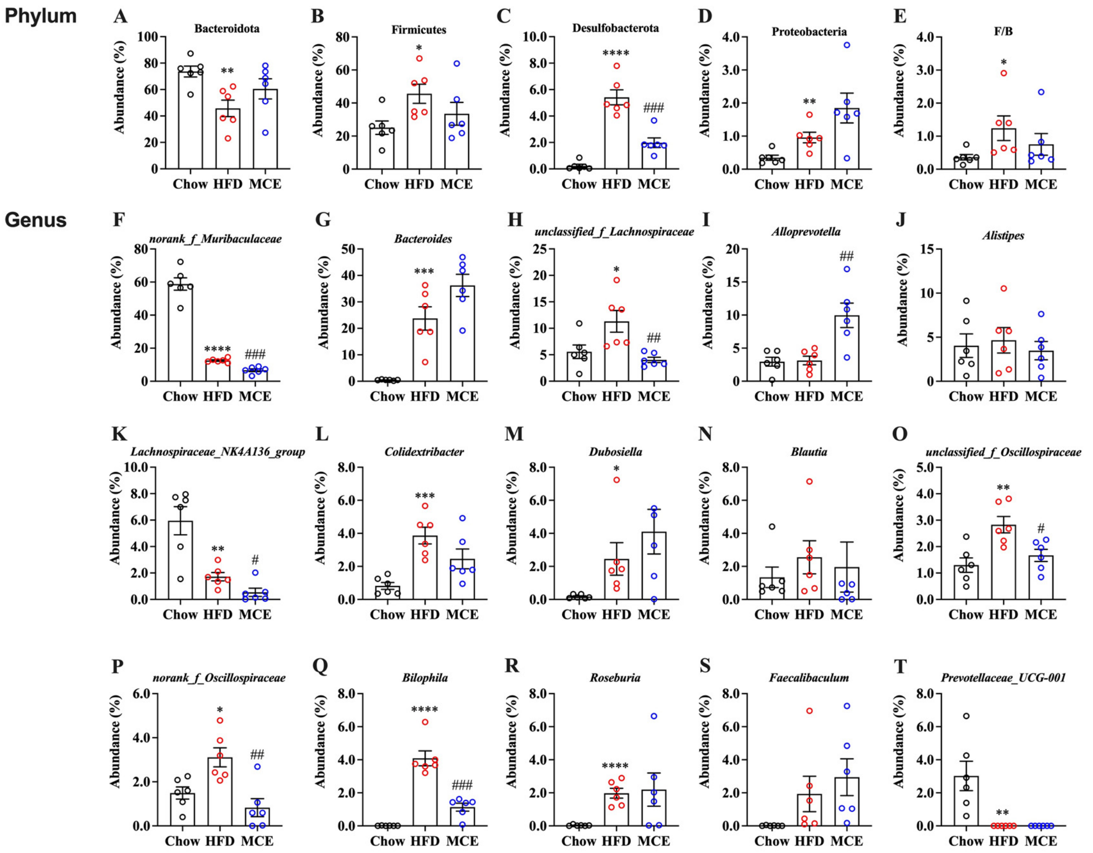

Figure S1. The relative abundance of gut microbiota at phylum (A-E) and genus (F-T) levels. **p* < 0.05 versus chow group, ***p* < 0.01 versus chow group, ****p* < 0.001 versus chow group, *****p* < 0.0001 versus chow group; $^{\#}p$ < 0.05 versus HFD group, $^{\#\#}p$ < 0.01 versus HFD group, $^{\#\#\#}p$ < 0.001 versus HFD group.
